# MITIGATING SOCIAL ISOLATION AND LONELINESS AMONG RESIDENTS IN SUBURBAN HOUSING COMPLEXES IN JAPAN

**DOI:** 10.1093/geroni/igae098.0672

**Published:** 2024-12-31

**Authors:** Keiko Katagiri, Nahyun Kim

**Affiliations:** Kobe University, Kobe, Hyogo, Japan; Kobe University, Kobe, Hyogo, Japan

## Abstract

Social isolation and loneliness represent significant societal challenges in Japan, particularly with the increasing number of older adults living alone. Comparative international surveys have highlighted weaker familial and neighborly ties among Japanese older adults, indicating a heightened risk of social isolation. In response, we initiated an action research, “Waigaya project” in Kobe, Japan, in 2022 to foster community connections and combat isolation among older suburban residents. The project comprises “Waigaya forums” and “Waigaya college.” Forums were events which aim to engage residents and promote participation, while “Waigaya college” offered ongoing educational opportunities to enhance knowledge and cultivate resilience for individuals facing extended lifespans. Over two forums and six college sessions, 103 participants aged 3 to 92 attended, with 25.7% attending both, 35.6% exclusively attending college, and 38.6% only participating in forums. College attendance varied, with 19.4% attending 4 to 6 times, 20.9% attending 2 to 3 times, and 59.7% attending once. Post-session surveys consistently showed high participant satisfaction and meaningful peer interactions, underscoring the project’s success. Notably, the project showed promising signs of identifying and nurturing potential core participants who may contribute to future community leadership, while also supporting the initial stages of developing a community network. The “Waigaya project” emerges as an effective strategy for addressing social isolation and loneliness among suburban residents in Japan, offering a promising model for similar initiatives worldwide. By prioritizing community engagement and lifelong learning, this project demonstrates the potential for meaningful impact in combatting social isolation and promoting well-being among older adults.

